# Supplementary material for: Acetate is a beneficial nutrient for E. coli at low glycolytic flux
Source: EMBO J. 2023 Jun 12;42(15):e113079. doi: 10.15252/embj.2022113079 (PMC10390867; doi:10.15252/embj.2022113079)
Supplement: Supplementary file 1 — Appendix [file EMBJ-42-e113079-s007.pdf]

Acetate is a beneficial nutrient for E. coli at low glycolytic flux

Pierre Millard, Thomas Gosselin-Monplaisir, Sandrine Uttenweiler-Joseph, Brice Enjalbert

Contains Appendix Table S1.

Primer name	Sequence 5′–3′
**pgi forward external**	CTTCCAAAGTCACAATTCTCAAAATCAGAAGAGT
**pgi reverse external**	TGAACGCCTTATCCGGCCTAC
**pgi forward internal**	ACTTCGATGAAATGAAAGACGTTACGATCG
**pfkA forward external**	TTGTTATACTATTTGCACATTCGTTGGATCACT
**pfkA reverse external**	CATCGGTTTCAGGGTAAAGGAATCTG
**pfkA forward internal**	ATGATTAAGAAAATCGGTGTGTTGACAAGC
**acs forward external**	TACAGGTTTTGCGGGAGCAGCCGTTT
**acs reverse external**	CCTCATGCAGGACTTCATTATTAAGACGGTC

Appendix Table S1. PCR primers used to check the *acs*, *pgi* and *pfkA* deletions.
