# Supplementary material for: Acetate is a beneficial nutrient for E. coli at low glycolytic flux
Source: EMBO J. 2023 Jun 12;42(15):e113079. doi: 10.15252/embj.2022113079 (PMC10390867; doi:10.15252/embj.2022113079)
Supplement: Supplementary file 2 — Expanded View Figures PDF [file EMBJ-42-e113079-s011.pdf]

Expanded View Figures

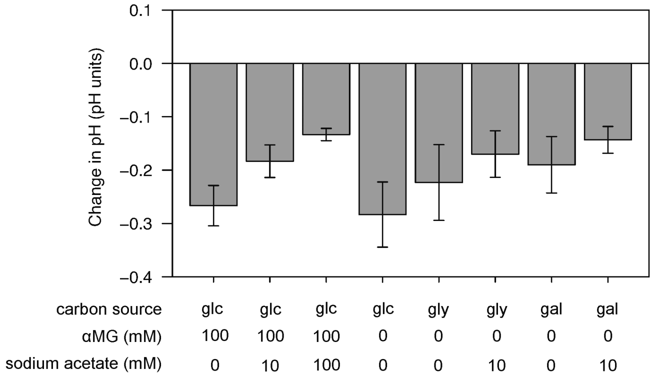

Figure EV1. Change in pH of the cultivation medium of *Escherichia coli* grown on glycolytic substrates plus different concentrations of αMG and acetate.

Change in pH of the cultivation medium during growth of *E. coli* K-12 BW25113 on glucose, glycerol, or galactose plus different concentrations of αMG and acetate. Change in pH was measured in the exponential growth phase where growth rates and extracellular fluxes were measured (OD between ~0.1 and ~1.5). Mean values and standard deviations (error bars) were estimated from three independent biological replicates.

Source data are available online for this figure.

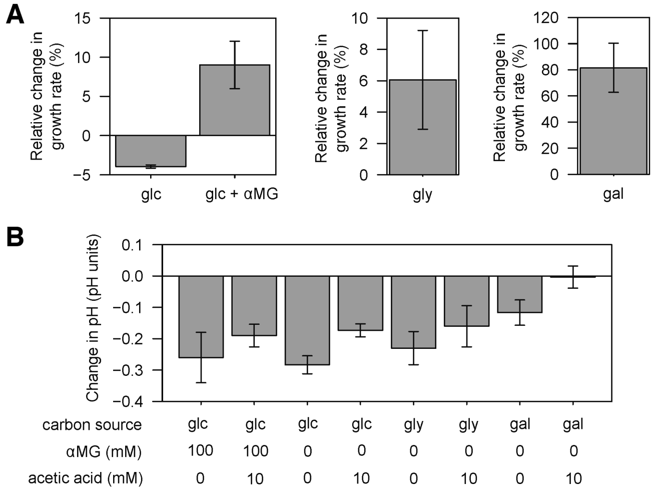

Figure EV2. Response of *Escherichia coli* to acetic acid.

A Relative change in the growth rate of *E. coli*K-12 BW25113 wild-type induced by the presence of 10 mM acetic acid during growth on glucose (without or with 100 mM αMG), glycerol, or galactose. Mean values and standard deviations (error bars) were estimated from three independent biological replicates.

B Change in pH of the cultivation medium during growth of *E. coli* K-12 BW25113 wild-type on glucose, glycerol, or galactose plus different concentrations of αMG and acetic acid. Change in pH was measured in the exponential growth phase where growth rates were measured (OD between ~0.1 and ~1.5). Mean values and standard deviations (error bars) were estimated from three independent biological replicates.

Source data are available online for this figure.

© 2023 The Authors
