# Supplementary material for: Acetate is a beneficial nutrient for E. coli at low glycolytic flux
Source: EMBO J. 2023 Jun 12;42(15):e113079. doi: 10.15252/embj.2022113079 (PMC10390867; doi:10.15252/embj.2022113079)
Supplement: Supplementary file 12 [file EMBJ-42-e113079-s004.pdf]

THE EMBO JOURNAL

Acetate is a beneficial nutrient for E. coli at low glycolytic flux

Pierre Millard, Thomas Gosselin-Monplaisir, Sandrine Uttenweiler-Joseph, and Brice Enjalbert
DOI: 10.15252/embj.2022113079

Corresponding author(s): Pierre Millard (millard@insa-toulouse.fr)

Review Timeline:

Submission Date:	16th Nov 22
Editorial Decision:	17th Jan 23
Revision Received:	31st Mar 23
Editorial Decision:	17th May 23
Revision Received:	23rd May 23
Accepted:	30th May 23

Editor: Ieva Gailite

Transaction Report:

(Note: With the exception of the correction of typographical or spelling errors that could be a source of ambiguity, letters and reports are not edited. Depending on transfer agreements, referee reports obtained elsewhere may or may not be included in this compilation. Referee reports are anonymous unless the Referee chooses to sign their reports.)

Dear Dr. Millard,

Thank you for submitting your manuscript for consideration by the EMBO Journal. I sincerely apologise for the protracted manuscript evaluation process due to the holiday period and delays in referee report submission. We have now received comments from two reviewers, which are included below for your information.

As you will see from the reports, both reviewers find the study of interest, while also indicating a number of concerns regarding the presented analysis that would have to be addressed before they can support publication here. From my side, I find the reviewers' comments reasonable. Therefore, based on these generally positive assessments, I invite you to address the issues raised by the reviewers in a revised manuscript. From the editorial side, please cite and discuss the published work on the co-utilization of gluconeogenic substrates including oxaloacetate when glycolytic flux is reduced (Okano et al, Nat. Microb. 2020).

I think it would be helpful to discuss the revision in more detail via email or phone/videoconferencing. I should also add that it is The EMBO Journal policy to allow only a single major round of revision and that it is therefore important to resolve the main concerns at this stage.

We generally allow three months as standard revision time. As a matter of policy, competing manuscripts published during this period will not negatively impact on our assessment of the conceptual advance presented by your study. However, please contact me as soon as possible upon publication of any related work to discuss the appropriate course of action. Should you foresee a problem in meeting this three-month deadline, please let us know in advance and we may be able to grant an extension.

When preparing your letter of response to the referees' comments, please bear in mind that this will form part of the Review Process File and will therefore be available online to the community. For more details on our Transparent Editorial Process, please visit our website: https://www.embopress.org/page/journal/14602075/authorguide#transparentprocess. Please also see the attached instructions for further guidelines on preparation of the revised manuscript.

Please feel free to contact me if you have any further questions regarding the revision. Thank you for the opportunity to consider your work for publication. I look forward to receiving the revised manuscript.

Yours sincerely,

Ieva Gailite

Ieva Gailite, PhD
Senior Scientific Editor
The EMBO Journal
Meyerhofstrasse 1
D-69117 Heidelberg
Tel: +4962218891309
i.gailite@embojournal.org

Instructions for preparing your revised manuscript:

Please make sure you upload a letter of response to the referees' comments together with the revised manuscript.

Please also check that the title and abstract of the manuscript are brief, yet explicit, even to non-specialists.

When assembling figures, please refer to our figure preparation guideline in order to ensure proper formatting and readability in print as well as on screen:
https://bit.ly/EMBOPressFigurePreparationGuideline
See also guidelines for figure legends: https://www.embopress.org/page/journal/14602075/authorguide#figureformat

At EMBO Press we ask authors to provide source data for the main manuscript figures. Our source data coordinator will contact you to discuss which figure panels we would need source data for and will also provide you with helpful tips on how to upload and organize the files.

IMPORTANT: When you send the revision we will require
- a point-by-point response to the referees' comments, with a detailed description of the changes made (as a word file).

- a word file of the manuscript text.
- individual production quality figure files (one file per figure)
- a complete author checklist, which you can download from our author guidelines (https://www.embopress.org/page/journal/14602075/authorguide).
- Expanded View files (replacing Supplementary Information)
Please see out instructions to authors
https://www.embopress.org/page/journal/14602075/authorguide#expandedview

Please remember: Digital image enhancement is acceptable practice, as long as it accurately represents the original data and conforms to community standards. If a figure has been subjected to significant electronic manipulation, this must be noted in the figure legend or in the 'Materials and Methods' section. The editors reserve the right to request original versions of figures and the original images that were used to assemble the figure.

Further information is available in our Guide For Authors: https://www.embopress.org/page/journal/14602075/authorguide

We realize that it is difficult to revise to a specific deadline. In the interest of protecting the conceptual advance provided by the work, we recommend a revision within 3 months (17th Apr 2023). Please discuss the revision progress ahead of this time with the editor if you require more time to complete the revisions.

Referee #1:

General
Authors describe a systems biology approach to decipher the co-consumption of acetate and glucose using an existing kinetic model of E coli metabolism. Specifically they aim to investigate the consumption of acetate under reduced glycolytic flux and demonstrate that reducing glycolytic flux results in co-utilization of acetate and glucose. They also tested two additional substrate with low glycolytic flux and show that co-utilization occurs under these conditions as well. The paper is well written and easy to read and this reviewer congratulates authors on well researched study. While this is an elegant study combining the use of existing model with some new data on E coli physiology, there are still several improvements required to this study as follows. In particular if the authors can show better comparison of the modeling results with data and also add in discussion, it will be valuable. The data showing reversible acetate flux is really an important contribution.

Major Revisions
1)Authors show in Figure 1 B that the glycolytic flux decreases as a function of the acetate concentrations. However, this reviewer feels this result can be attributed to the assumption made in the model (see below) where the inhibition is included without any clear mechanism behind it. Should this be true authors should highlight and explain how this can happen at a molecular level and not use this modeling result in 1B which is trivial given the equation below.
vglycolysis=Vmaxglycolysis·GLCGLC+Km_GLC ⎡⎣⎢⎢·11+ACEenvKi_ACE⎤⎦⎥⎥
This is a key weakness of the paper. Could this not be an explanation for potential toxicity when the acetate concentrations increase to the point at which glucose uptake rate is lowered and this decrease cannot be compensated by the increase in acetate uptake rate. So authors should plot Figure 1 to higher acetate concentrations and show how the graphs look.

2) Furthermore, the authors show that in Figure 1 C the reversal of acetate flux occurs over a range of glycolytic flux. The reversal is clearly due to the thermodynamic term in the kinetics and as the ACEEnv increases the VAcetate_Exchange and, ACKA, PTA is negative resulting in higher ACCOA and increased growth that can overcome the reduction in ACCOA due to glycolysis. This explanation would have been useful to add and of course seems trivial given the modeling result. One of the questions is around the use of the thermodynamic terms especially around the Keq term. While this Keq can be obtained from the standard Gibbs free energy of the reaction for the ACKA, PTA reactions, it is not clear how the transport/exchange Keq was calculated. This parameter is not explained even in their previous eLife paper and deserves additional mention especially because this parameter clearly influences the results in the Figure 1C and 1D. It will be helpful also to do a sensitivity analysis over the range of uncertainty in exchange Keq given that the thermodynamics of this term is unlikely to be inferred accurately.

3) In Figure 2 authors describe some nice experimental data that provides significant support for their claim regarding the reversible nature of the acetate flux and its relation to both the acetate concentration in the environment and the glycolytic flux. The data visualization could be improved by using just open symbols as opposed to open symbols with ligher shading, But what was strikingly absent is the model predictions. Authors should the model predictions on the same plots.
4) In Figure 2 can the authors also plot the growth rate with the total carbon uptake to support their claim that the growth rate is

not impacted by the fact that the total carbon uptake remains constant. Can the authors also show the modeling data from Figure 1 relating the total carbon flux and the growth rate corresponding to figure 1C 1D ?
5) Figure 3 Authors should plot the Figure 1 C data where the line separating the positive and negative acetate exchange flux determines the threshold acetate concentration at which acetate flux reverses for a given glucose uptake and compare it with the data in Figure 3. Even if the data does not match well with the modeling results I think it is very useful to understand if the trends are similar in more clearer manner than what the authors are showing so far in this paper.
6) Again like Figure 3 authors can compare the model predictions of the growth rate as a function of the acetate concentration at maximal glucose uptake and 20% value along with the experimental data in Figure 4. Also, please add error bars in the case with the reduced glycolytic flux to ensure that the differences between the low and high acetate concentration cases are more apparent.
7) Another important consideration in looking at acetate toxicity is the pH. Did the authors adjust the pH to 7 during the growth experiments ? If so this should be mentioned in the discussion. If not given that this is not part of the model, authors should add a disclaimer. In particular, methods mention that the authors used sodium acetate at 100mM, which of course is not the same as the toxicity when acetic acid is produced especially in a biotechnology concept, a point worth discussing.

Referee #2:

The utilization of diverse carbon sources makes microbial strains survive in various environments and more attractive when applied in biotechnology including metabolic engineering. It has been thought that acetate is a useless by-product because several previous studies have validated its toxicity during cellular metabolism. However, in this study, the authors showed that acetate can be utilized as one of the alternative carbon sources depending on the glycolytic activity. In spite of the impressive work they indicated, some points are required or edited to clarify the points mentioned in this study.

Major points
1. The authors showed that the metabolic direction of acetate (production or consumption) can be changed depending on the glycolytic activity and acetate concentration. Especially, they downregulated the glycolytic flux by adding the glycolytic inhibitor, αMG. However, in case of the biotechnological application such as metabolic engineering, the glycolytic flux was inhibited by knocking out or down of genes related to glycolysis. So, how about showing the data indicating the interplay between glycolytic substrate and acetate by knocking out major genes (e.g. ptsG or ptsH) related in glycolysis?

2. The authors displayed the data related to the co-consumption of glucose and acetate. And they mentioned the potential of acetate as the carbon source for cell growth. Then, how about the data when using acetate as a sole carbon source? It might be better to support their viewpoint if they suggest the data.

3. The authors mentioned that acetate can be applied as a beneficial nutrient for E. coli, not just a toxic waste. They said that there is a switch between acetate production and consumption and it was dependent on glycolytic flux and acetate concentration. However, even though they showed the value point of the switch, it looks insufficient why the reversal occurs. It might be better to support them by discussing it in more detailedly, especially in terms of metabolic regulation inside E. coli.

Minor points
1. Mistyping on result 'At low glycolytic flux, acetate activates E. coli metabolism and growth at the transcriptional level'. On 2nd line of 2nd paragraph (Figure 6A → Figure 5A) and 3rd line of 3rd paragraph (Figure 6B → Figure 5B) of the result, respectively.

We provide the revised manuscript with tracked changes along with this point-by-point response to the editorial and referees' comments.

Note: Editorial and referees' comments are in black, our responses are in blue.

Response to Editorial comment

From the editorial side, please cite and discuss the published work on the co-utilization of gluconeogenic substrates including oxaloacetate when glycolytic flux is reduced (Okano et al, Nat. Microb. 2020).

In their very interesting work, Okano et al. showed that oxaloacetate boosts *E. coli* growth on glycolytic substrates (glycerol, xylose and fucose). Consistently with these observations, they suggested that gluconeogenic substrates enhance *E. coli* growth on glycolytic substrates, and they built a mathematical model that supports their theory. We show that acetate indeed boosts *E. coli* growth at low glycolytic flux, but it also inhibits growth at high glycolytic flux. In other words, Okano et al.'s theory – and the underlying model – does not capture the non-monotonic growth response of *E. coli* to acetate uncovered in our study. We discuss Okano et al.'s work in the revised manuscript:

"Likewise, the recent suggestion that gluconeogenic substrates such as acetate enhance E. coli growth on glycolytic substrates (as observed for mixtures of oxaloacetate with glycerol, xylose or fucose) (Okano, et al., 2020) also fails to capture the non-monotonic growth response of E. coli to acetate."

Response to Referees' comments

Referee #1:

General

Authors describe a systems biology approach to decipher the co-consumption of acetate and glucose using an existing kinetic model of E coli metabolism. Specifically they aim to investigate the consumption of acetate under reduced glycolytic flux and demonstrate that reducing glycolytic flux results in co-utilization of acetate and glucose. They also tested two additional substrate with low glycolytic flux and show that co-utilization occurs under these conditions as well. The paper is well written and easy to read and this reviewer congratulates authors on well researched study. While this is an elegant study combining the use of existing model with some new data on E coli physiology, there are still several improvements required to this study as follows. In particular if the authors can show better comparison of the modeling results with data and also add in discussion, it will be valuable. The data showing reversible acetate flux is really an important contribution.

We thank referee 1 for their positive feedback and their interest in this work. We have modified the manuscript to address all the points they raise. Regarding the modelling aspects of this work, i) we have performed a global sensitivity analysis to estimate the confidence intervals for the model predictions, and ii) we have added a better comparison of the modelling results with experimental data. Results confirm that the model predictions are robust to parameter uncertainty and are

consistent with experimental data, strengthening our conclusions. We have also carried out new experiments that demonstrate that the beneficial effect of acetate is not due to changes in pH and is maintained when using acetic acid instead of sodium acetate. These results also confirm the main findings of the study.

Major Revisions

1) Authors show in Figure 1 B that the glycolytic flux decreases as a function of the acetate concentrations. However, this reviewer feels this result can be attributed to the assumption made in the model (see below) where the inhibition is included without any clear mechanism behind it. Should this be true authors should highlight and explain how this can happen at a molecular level and not use this modeling result in 1B which is trivial given the equation below.

$$v_{glycolysis} = \frac{Vmax_{glycolysis} \cdot GLC}{GLC + Km\_GLC} \left[\cdot \frac{1}{1 + \frac{ACE_{env}}{Ki\_ACE}} \right]$$

This is a key weakness of the paper. Could this not be an explanation for potential toxicity when the acetate concentrations increase to the point at which glucose uptake rate is lowered and this decrease cannot be compensated by the increase in acetate uptake rate. So authors should plot Figure 1 to higher acetate concentrations and show how the graphs look.

We agree with referee 1 that the decrease of glucose uptake with increased acetate concentration is expected from the structure of the model. There is another inhibitory term in the model, which represents the effect of acetate on the TCA cycle and growth machinery. These inhibition terms – and the mechanisms they represent – are extensively discussed in the 2021 eLife paper and associated review reports (https://elifesciences.org/articles/63661#sa2), and their functional relevance has been validated experimentally. Importantly, despite the dual inhibitory effects of acetate on catabolism (e.g. glycolysis and TCA cycle) and anabolism (e.g. glutamate metabolism and growth machinery), the model predicts that increasing the concentration of acetate, which has been considered a toxic by-product for decades, should enhance *E. coli* growth at low glycolytic flux (Figure 1 and 4A). This *ab initio*, model-driven hypothesis, which is counterintuitive and far from trivial, was the main driver of this study. We have clarified and stressed these points in the revised manuscript (see Results, section "*Model-driven hypotheses on the interplay between glucose and acetate metabolism in Escherichia coli*.").

2) Furthermore, the authors show that in Figure 1 C the reversal of acetate flux occurs over a range of glycolytic flux. The reversal is clearly due to the thermodynamic term in the kinetics and as the ACEEnv increases the VAcetate_Exchange and, ACKA, PTA is negative resulting in higher ACCOA and increased growth that can overcome the reduction in ACCOA due to glycolysis. This explanation would have been useful to add and of course seems trivial given the modeling result.

For the sake of clarity, we have added this explanation (which does indeed seem trivial given the modelling results) in the Discussion section of the revised manuscript:

"In keeping with this hypothesis, our results support the concept of acetate flux being thermodynamically controlled by acetate itself, as detailed in (Enjalbert, et al., 2017), and we show that the thermodynamically-driven flux reversal is also controlled by the glycolytic flux. The reduction in glucose-derived acetyl-CoA is counterbalanced by an increased contribution of acetate, which sustains growth."

One of the questions is around the use of the thermodynamic terms especially around the Keq term. While this Keq can be obtained from the standard Gibbs free energy of the reaction for the ACKA, PTA reactions, it is not clear how the transport/exchange Keq was calculated. This parameter is not explained even in their previous eLife paper and deserves additional mention especially because this parameter clearly influences the results in the Figure 1C and 1D. It will be helpful also to do a sensitivity analysis over the range of uncertainty in exchange Keq given that the thermodynamics of this term is unlikely to be inferred accurately.

We agree and are grateful for this suggestion. In our previous article in which the model is described, the choice of the Keq value (set to 1) was based on the assumption that "Intracellular and extracellular acetate concentrations equilibrate over time" (see *Table 2. Values of kinetic parameters*.). Still, we agree with referee 1 that this value is unlikely to be inferred accurately. To address this important question, we evaluated the effects of parameter uncertainties on all the predictions of the model using global sensitivity analysis (Monte Carlo analysis), as detailed in the "Materials and methods" section of the revised manuscript. Importantly, we varied the values of *all* estimated model parameters, not just the Keq term, and we estimated the standard deviations of all predicted values (shaded areas in Figures 2A-D, 3A-B and 4A). These new results confirm that all the predictions of the model are robust to parameter uncertainty.

3) In Figure 2 authors describe some nice experimental data that provides significant support for their claim regarding the reversible nature of the acetate flux and its relation to both the acetate concentration in the environment and the glycolytic flux. The data visualization could be improved by using just open symbols as opposed to open symbols with ligher shading, But what was strikingly absent is the model predictions. Authors should the model predictions on the same plots.

In the revised version of Figure 2, the data collected in the absence and presence of acetate are represented by different symbols and we use lighter shading to facilitate comparisons of the fitted lines. We have made similar changes to Figures 3 and 4. We thank referee 1 for this suggestion, which improves the clarity of the figures.

The modelling data shown in Figure 1 were obtained by (computationally) varying the glycolytic activity (i.e. Vmax), while Figure 2 was obtained by (experimentally) varying the concentration of glycolytic inhibitor. Though conceptually similar (i.e. increasing the concentration of glycolytic inhibitor reduces the glycolytic activity, as confirmed experimentally), this difference precludes direct quantitative comparisons between the simulated data (Figure 1) and the experimental data (Figures 2-4) on the same plots. Still, we agree that facilitating visual comparisons between the predictions of the model (Figure 1) and experimental data would strengthen our results and have followed referee 1's suggestion (comment #5). In the revised manuscript, we compare the predicted profiles with the measured profiles in Figures 2, 3 and 4. These are consistent with each other and confirm that the trends are similar.

4) In Figure 2 can the authors also plot the growth rate with the total carbon uptake to support their claim that the growth rate is not impacted by the fact that the total carbon uptake remains constant.

Since both the growth rate and the total carbon uptake remain stable in the presence of acetate despite a ~2-fold change in glucose uptake (Figures 2A and 2D of the original manuscript), no clear relationship can be established between these two variables in the presence of acetate, and the corresponding plot is not informative (see below).

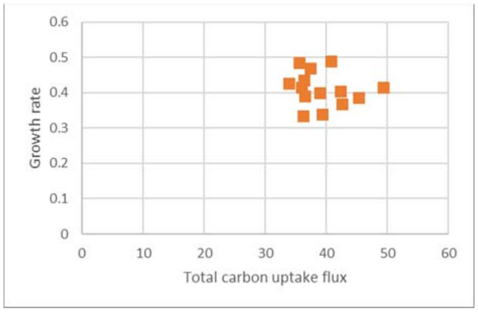

In the corresponding paragraph, we investigate the control exerted by glycolysis on *E. coli* metabolism in the presence or absence of acetate, which was done by quantifying the response of *E. coli* to a modulation of the glycolytic flux. The key message is that both the growth rate and the total carbon uptake were less sensitive to a reduction of glucose uptake in the presence of acetate than in the absence of acetate (panels C-D and G-H of the revised manuscript). This indicates that acetate compensates for the drop in carbon uptake caused by the reduction in glucose uptake (as confirmed by Figures 2K-L of the revised manuscript). We have also added new panels to support these claims (Figures 2I-J) and have rephrased this paragraph in the revised manuscript (see paragraph "*Enhanced acetate utilization at low glycolytic flux buffers carbon uptake*").

Can the authors also show the modeling data from Figure 1 relating the total carbon flux and the growth rate corresponding to figure 1C 1D ?

We now compare the predicted total carbon flux and growth rates to experimental data in Figure 2 of the revised manuscript (panels C and G for the growth rate, panels D and H for the total carbon flux).

5) Figure 3 Authors should plot the Figure 1 C data where the line separating the positive and negative acetate exchange flux determines the threshold acetate concentration at which acetate flux reverses for a given glucose uptake and compare it with the data in Figure 3. Even if the data does not match well with the modeling results I think it is very useful to understand if the trends are similar in more clearer manner than what the authors are showing so far in this paper.

As suggested by referee 1, we compare the modelling data for the threshold acetate concentration (Figure 1C) to experimental measurements in Figure 3 of the revised manuscript (new panels A, B and D). The simulated and experimental profiles are similar.

6) Again like Figure 3 authors can compare the model predictions of the growth rate as a function of the acetate concentration at maximal glucose uptake and 20% value along with the experimental data in Figure 4.

We compare the predicted growth rates with experimental data in Figure 4 of the revised manuscript, as suggested by referee 1. The predicted profiles are consistent with the experimental profiles.

Also, please add error bars in the case with the reduced glycolytic flux to ensure that the differences between the low and high acetate concentration cases are more apparent.

Each dot was obtained from independent experiments performed at different acetate concentrations, therefore biological variability is represented by the dispersion of the points around the corresponding lines. To highlight the differences between low and high acetate concentration cases in the revised manuscript, we have performed t-tests on the difference in growth rates

measured at low (< 0.5 mM) and high (60 mM) acetate concentrations (data from Figure 4B in the revised manuscript). We have also performed t-tests to determine the statistical significance of the change in growth rate in the absence of acetate vs in the presence of 10 mM acetate (Figures 4C-D, 6 and 7). The differences between the values measured at low and high acetate concentrations are statistically significant (p values between 3.10^{-5} and 1.10^{-2}). We have added this new information to the corresponding paragraphs of the Results section (see paragraphs "*Acetate enhances E. coli growth at low glycolytic flux.*", "*Acetate-enhanced growth is supported by the Pta-AckA pathway.*", and "*Acetate boosts growth of E. coli on glycerol and galactose.*")

7) Another important consideration in looking at acetate toxicity is the pH. Did the authors adjust the pH to 7 during the growth experiments ? If so this should be mentioned in the discussion. If not given that this is not part of the model, authors should add a disclaimer.

We did not adjust the pH during the growth experiments, and we agree with referee 1 that pH could play an important role in acetate toxicity. To address this important point, we have carried out new experiments to measure the change in pH during the growth. Because of the high buffer capacity of the M9 cultivation medium, the pH changed by less than 0.3 pH units in virtually all experiments, and in most experiments by less than 0.2 pH units. These results indicate that the metabolic and physiological responses of *E. coli* identified in this work are not due to changes in pH. We have added these new data in the revised manuscript and discuss pH variations in the Discussion section:

"Previous reports have shown that the "toxicity" of acetate for E. coli growth depends on the pH (Lawford and Rousseau, 1993; Orr, et al., 2019; Pinhal, et al., 2019). In our experiments, the pH was initially set to 7.0 but was not adjusted during growth. Nevertheless, the high buffer capacity of the M9 cultivation medium ensured that the pH remained stable in the exponential growth phase where growth rates and extracellular fluxes were measured, with changes of less than 0.3 pH unit in all experiments (Figure EV1). Therefore, the metabolic and physiological responses of E. coli to acetate highlighted in this study are not due to changes in pH."

In particular, methods mention that the authors used sodium acetate at 100mM, which of course is not the same as the toxicity when acetic acid is produced especially in a biotechnology concept, a point worth discussing.

We agree and to address this important point, we have carried out additional experiments to test the impact on growth of acetic acid (with the pH set to 7.0 by addition of NH_4OH) instead of sodium acetate. The results of these experiments confirm and strengthen those obtained with sodium acetate. These data are included and discussed in the revised manuscript:

"Another potential issue is that the effect of acetate might also depend on the concentration of salts (Bae and Lee, 2017; Lee, et al., 2010). The effect of acetate added as sodium salt may differ from its effect when added as acetic acid. However, repeat experiments performed with acetic acid instead of sodium acetate showed that the beneficial effect of acetate on E. coli growth on glucose, galactose and glycerol was maintained (Figure EV2)."

Referee #2:

The utilization of diverse carbon sources makes microbial strains survive in various environments and more attractive when applied in biotechnology including metabolic engineering. It has been thought that acetate is a useless by-product because several previous studies have validated its toxicity during cellular metabolism. However, in this study, the authors showed that acetate can be utilized

as one of the alternative carbon sources depending on the glycolytic activity. In spite of the impressive work they indicated, some points are required or edited to clarify the points mentioned in this study.

We thank referee 2 for their feedback and relevant suggestions. In particular, we have carried out new experiments using mutant strains with reduced glycolytic flux. These results confirm our findings and increase the value of this work.

Major points

1. The authors showed that the metabolic direction of acetate (production or consumption) can be changed depending on the glycolytic activity and acetate concentration. Especially, they downregulated the glycolytic flux by adding the glycolytic inhibitor, αMG. However, in case of the biotechnological application such as metabolic engineering, the glycolytic flux was inhibited by knocking out or down of genes related to glycolysis. So, how about showing the data indicating the interplay between glycolytic substrate and acetate by knocking out major genes (e.g. ptsG or ptsH) related in glycolysis?

In the original manuscript, we used two different strategies to modulate the glycolytic flux and experimentally test our model-driven hypotheses: i) using an inhibitor and ii) changing the carbon source. We thank referee 2 for suggesting a third, orthogonal strategy, *i.e.* using mutant strains deleted for genes involved in glycolysis (which should also be valuable in biotechnological applications). Knocking out *ptsG* or *ptsH* would not significantly reduce the glycolytic flux (by only about 20 % according to Fuentes *et al*., 2013), but Long et al. (2019) describe mutant strains with much lower glycolytic flux. We grew the Δ*pgi* and Δ*pfkA* strains and tested their response to the presence of acetate in the absence of inhibitor. Acetate had a positive effect on growth in both strains, confirming our initial findings. We have added a paragraph in the results section of the revised manuscript:

"As an orthogonal, alternative strategy to the use of a glycolytic inhibitor, we tested the impact of acetate on the growth of mutant strains with low glycolytic flux, namely the E. coli K-12 BW25113 Δpgi and ΔpfkA strains, where the glycolytic flux is 24 and 29 % that of the wild-type strain, respectively (Long and Antoniewicz, 2019). Their growth rates were measured on 15 mM glucose, with or without 10 mM acetate and the effect of acetate was quantified as the relative change in growth rate. The wild-type isogenic strain was grown in the absence and presence of αMG (100 mM) as a control. As expected, acetate reduced the growth rate of the wild-type strain in the absence of αMG (−6 ± 1 %, p-value = 4.10^{-5}) but increased its growth in the presence of 100 mM αMG (+9 ± 2 %, p-value = 3.10^{-5}) (Figure 4C). The beneficial effect of acetate on growth was also observed in the Δpgi and ΔpfkA strains (Figure 4D), whose growth rate strongly increased despite the absence of αMG (Δpgi : +60 ± 19 %, p-value = 3.10^{-2}; ΔpfkA : +117 ± 21 %, p-value = 1.10^{-2}). These results confirm that the beneficial effect of acetate is not due to the presence of αMG but is a consequence of the decrease in glycolytic flux.

Overall, all results are in line with the predictions of the model and demonstrate that acetate enhances E. coli growth when the glycolytic flux is reduced by either chemical or genetic perturbations."

2. The authors displayed the data related to the co-consumption of glucose and acetate. And they mentioned the potential of acetate as the carbon source for cell growth. Then, how about the data

when using acetate as a sole carbon source? It might be better to support their viewpoint if they suggest the data.

The main objective of this work is to investigate the mutual regulation of glycolytic and acetate metabolism, starting from the idea that acetate has long been considered an inhibitor of *E. coli* growth on glycolytic substrates. Hence, we think that investigating *E. coli* growth on acetate as sole carbon source (*i.e*. in the absence of glycolytic substrate) would not provide additional insights into the interplay between glycolytic and acetate metabolisms. We feel that investigations of *E. coli* metabolism on acetate as sole carbon source have already been carried out (see *e.g.* Zhao and Shimizu 2003; Millard *et al*., 2014) and fall outside the scope of this study.

3. The authors mentioned that acetate can be applied as a beneficial nutrient for E. coli, not just a toxic waste. They said that there is a switch between acetate production and consumption and it was dependent on glycolytic flux and acetate concentration. However, even though they showed the value point of the switch, it looks insufficient why the reversal occurs. It might be better to support them by discussing it in more detailedly, especially in terms of metabolic regulation inside E. coli.

We mention in the introduction that "*The switch between acetate production and consumption was found to be determined thermodynamically by the acetate concentration itself*", as extensively tested and discussed in Enjalbert *et al.*, 2017. In this previous publication, we explain in detail why the flux reversal occurs. Here, we show that this thermodynamically-driven flux reversal, which is responsible for the growth boost, is also controlled by the glycolytic flux. We have made this point clearer in the Discussion section of the revised manuscript:

"In keeping with this hypothesis, our results support the concept of acetate flux being thermodynamically controlled by acetate itself, as detailed in (Enjalbert, et al., 2017), and we show that the thermodynamically-driven flux reversal is also controlled by the glycolytic flux. The reduction in glucose-derived acetyl-CoA is counterbalanced by an increased contribution from acetate, which sustains growth. Metabolic regulation is thus (at least partly) responsible for the interplay between acetate and glycolytic flux."

Minor points

1. Mistyping on result 'At low glycolytic flux, acetate activates E. coli metabolism and growth at the transcriptional level'. On 2nd line of 2nd paragraph (Figure 6A → Figure 5A) and 3rd line of 3rd paragraph (Figure 6B → Figure 5B) of the result, respectively.

This typo has been corrected in the revised manuscript.

Dear Pierre,

Thank you for submitting a revised version of your manuscript. I sincerely apologise for the protracted assessment process due to delays in the submission of referee reports. Your study has been seen by one of the original referees, who finds that their previous concerns have been addressed and now recommends publication of the manuscript. There also remain a few editorial points that have to be addressed before I can extend formal acceptance of the manuscript:

1. Our publisher has done their pre-publication check on your manuscript. I have attached the file here. Please take a look at the word file and the comments regarding the figure legends and respond to the issues. Please also use this version when you resubmit the revised version.
2. Figure panels 7A and 7B are not mentioned in the manuscript text.
3. CRediT has replaced the traditional author contributions section because it offers a systematic, machine-readable author contributions format that allows for more effective research assessment. Please remove the Authors Contributions from the manuscript and use the free text boxes beneath each contributing author's name in our online submission system to add specific details on the author's contribution. More information is available in our guide to authors.
4. I would like to propose a couple of minor changes in the article title, synopsis and abstract to increase their accessibility to our more general readership. I have also written a short blurb that will accompany the title of your manuscript in our online table of contents. Please take a look at the text below and in the attached manuscript text file and let me know if any corrections are needed.

Title:
Acetate metabolism boosts E. coli growth at low glycolytic flux

Blurb:
Systems biology analysis highlights an unexpected role for acetate as a compensatory carbon source under conditions of reduced glycolysis.

Synopsis:
Acetate is traditionally viewed as a toxic by-product of glycolytic metabolism that inhibits microbial growth. This study demonstrates that acetate can act as a co-substrate of glycolytic nutrients and promote E. coli growth when the glycolytic flux is low.
- Glycolytic flux determines the role of acetate as a by-product or co-substrate of glycolytic nutrients.
- Acetate utilization by the Pta-AckA pathway is enhanced at reduced glycolytic flux.
- Acetate promotes bacterial growth at low glycolytic flux.
- Acetate increases the robustness of E. coli responses to glycolytic perturbations by buffering carbon uptake.

Please let me know if you have any further questions regarding any of these points. You can use the link below to upload the revised files.

Thank you again for giving us the chance to consider your manuscript for The EMBO Journal. I look forward to receiving the final version.

With best regards,

Ieva

Ieva Gailite, PhD
Senior Scientific Editor
The EMBO Journal
Meyerhofstrasse 1
D-69117 Heidelberg
Tel: +4962218891309
i.gailite@embojournal.org

Further information is available in our Guide For Authors: https://www.embopress.org/page/journal/14602075/authorguide

We realize that it is difficult to revise to a specific deadline. In the interest of protecting the conceptual advance provided by the work, we recommend a revision within 3 months (15th Aug 2023). Please discuss the revision progress ahead of this time with the editor if you require more time to complete the revisions.

Referee #2:

Authors addressed all the comments raised by the reviewers in the revised version. Current version of manuscript is acceptable for publication in EMBO Journal.

The authors have addressed all minor editorial requests.

2nd Revision - Editorial Decision **30th May 2023**

Dear Pierre,

Thank you for addressing the final minor editorial points. I apologise for the delay due to my absence from the office last week. I am now pleased to inform you that your manuscript has been accepted for publication in the EMBO Journal.

Please note that it is EMBO Journal policy for the transcript of the editorial process (containing referee reports and your response letter) to be published as an online supplement to each paper. If you do NOT want this, you will need to inform the Editorial Office via email immediately. More information is available here:
https://www.embopress.org/page/journal/14602075/authorguide#transparentprocess

Your manuscript will be processed for publication in the journal by EMBO Press. Manuscripts in the PDF and electronic editions of The EMBO Journal will be copy edited, and you will be provided with page proofs prior to publication. Please note that supplementary information is not included in the proofs.

You will be contacted by Wiley Author Services to complete licensing and payment information. The required 'Page Charges Authorization Form' is available here: https://www.embopress.org/pb-assets/embo-site/tej_apc.pdf - please download and complete the form and return to embopressproduction@wiley.com

EMBO Press participates in many Publish and Read agreements that allow authors to publish Open Access with reduced/no publication charges. Check your eligibility: https://authorservices.wiley.com/author-resources/Journal-Authors/open-access/affiliation-policies-payments/index.html

Should you be planning a Press Release on your article, please get in contact with embojournal@wiley.com as early as possible, in order to coordinate publication and release dates.

If you have any questions, please do not hesitate to call or email the Editorial Office. Thank you again for this contribution to The EMBO Journal and congratulations on a successful publication!

Best regards,

Ieva

Ieva Gailite, PhD
Senior Scientific Editor
The EMBO Journal
Meyerhofstrasse 1
D-69117 Heidelberg
Tel: +4962218891309
i.gailite@embojournal.org

EMBO Press Author Checklist

Corresponding Author Name: Pierre Millard	
Journal Submitted to: EMBO Journal	
Manuscript Number: EMBOJ-2022-113079	

USEFUL LINKS FOR COMPLETING THIS FORM
The EMBO Journal - Author Guidelines
EMBO Reports - Author Guidelines
Molecular Systems Biology - Author Guidelines
EMBO Molecular Medicine - Author Guidelines

Reporting Checklist for Life Science Articles (updated January
This checklist is adapted from Materials Design Analysis Reporting (MDAR) Checklist for Authors. MDAR establishes a minimum set of requirements in transparent reporting in the life sciences (see Statement of Task: 10.31222/osf.io/9sm4x). Please follow the journal's guidelines in preparing your
Please note that a copy of this checklist will be published alongside your article.

Abridged guidelines for figures
1. Data
The data shown in figures should satisfy the following conditions:
- the data were obtained and processed according to the field's best practice and are presented to reflect the results of the experiments in an accurate and unbiased manner.
- ideally, figure panels should include only measurements that are directly comparable to each other and obtained with the same assay.
- plots include clearly labeled error bars for independent experiments and sample sizes. Unless justified, error bars should not be shown for technical
- if n<5, the individual data points from each experiment should be plotted. Any statistical test employed should be justified.
- Source Data should be included to report the data underlying figures according to the guidelines set out in the authorship guidelines on Data

2. Captions
Each figure caption should contain the following information, for each panel where they are relevant:
- a specification of the experimental system investigated (eg cell line, species name).
- the assay(s) and method(s) used to carry out the reported observations and measurements.
- an explicit mention of the biological and chemical entity(ies) that are being measured.
- an explicit mention of the biological and chemical entity(ies) that are altered/varied/perturbed in a controlled manner.
- the exact sample size (n) for each experimental group/condition, given as a number, not a range;
- a description of the sample collection allowing the reader to understand whether the samples represent technical or biological replicates (including how many animals, litters, cultures, etc.).
- a statement of how many times the experiment shown was independently replicated in the laboratory.
- definitions of statistical methods and measures:

  - common tests, such as t-test (please specify whether paired vs. unpaired), simple χ2 tests, Wilcoxon and Mann-Whitney tests, can be unambiguously identified by name only, but more complex techniques should be described in the methods section;
  - are tests one-sided or two-sided?
  - are there adjustments for multiple comparisons?
  - exact statistical test results, e.g., P values = x but not P values < x;
  - definition of 'center values' as median or average;
  - definition of error bars as s.d. or s.e.m.

Please complete ALL of the questions below.
Select "Not Applicable" only when the requested information is not relevant for your study.

Materials

Newly Created Materials	Information included in the manuscript?	In which section is the information available? (Reagents and Tools Table, Materials and Methods, Figures, Data Availability Section)
New materials and reagents need to be available; do any restrictions apply?	Not Applicable	

Antibodies	Information included in the manuscript?	In which section is the information available? (Reagents and Tools Table, Materials and Methods, Figures, Data Availability Section)
For **antibodies** provide the following information:<br>- Commercial antibodies: RRID (if possible) or supplier name, catalogue number and or/clone number<br>- Non-commercial: RRID or citation	Not Applicable	

DNA and RNA sequences	Information included in the manuscript?	In which section is the information available? (Reagents and Tools Table, Materials and Methods, Figures, Data Availability Section)
**Short novel DNA or RNA including primers, probes**: provide the sequences.	Yes	Materials and Methods, Appendix

Cell materials	Information included in the manuscript?	In which section is the information available? (Reagents and Tools Table, Materials and Methods, Figures, Data Availability Section)
**Cell lines:** Provide species information, strain. Provide accession number in repository **OR** supplier name, catalog number, clone number, and/**OR** RRID.	Not Applicable	
**Primary cultures:** Provide species, strain, sex of origin, genetic modification status.	Not Applicable	
Report if the cell lines were recently **authenticated** (e.g., by STR profiling) and tested for mycoplasma contamination.	Not Applicable	

Experimental animals	Information included in the manuscript?	In which section is the information available? (Reagents and Tools Table, Materials and Methods, Figures, Data Availability Section)
**Laboratory animals or Model organisms:** Provide species, strain, sex, age, genetic modification status. Provide accession number in repository **OR** supplier name, catalog number, clone number, **OR** RRID.	Not Applicable	
**Animal observed in or captured from the field:** Provide species, sex, and age where possible.	Not Applicable	
Please detail **housing and husbandry conditions**.	Not Applicable	

Plants and microbes	Information included in the manuscript?	In which section is the information available? (Reagents and Tools Table, Materials and Methods, Figures, Data Availability Section)
**Plants:** provide species and strain, ecotype and cultivar where relevant, unique accession number if available, and source (including location for collected wild specimens).	Not Applicable	
**Microbes:** provide species and strain, unique accession number if available, and source.	Yes	Materials and Methods

Human research participants	Information included in the manuscript?	In which section is the information available? (Reagents and Tools Table, Materials and Methods, Figures, Data Availability Section)
If collected and within the bounds of privacy constraints report on age, sex and gender or ethnicity for all study participants.	Not Applicable	

Core facilities	Information included in the manuscript?	In which section is the information available? (Reagents and Tools Table, Materials and Methods, Figures, Data Availability Section)
If your work benefited from core facilities, was their service mentioned in the acknowledgments section?	Yes	Acknowledgements

Design

Study protocol	Information included in the manuscript?	In which section is the information available? (Reagents and Tools Table, Materials and Methods, Figures, Data Availability Section)

If study protocol has been **pre-registered, provide DOI in the manuscript**. For clinical trials, provide the trial registration number **OR** cite DOI.	Not Applicable	
Report the **clinical trial registration number** (at ClinicalTrials.gov or equivalent), where applicable.	Not Applicable	

Laboratory protocol	Information included in the manuscript?	In which section is the information available? (Reagents and Tools Table, Materials and Methods, Figures, Data Availability Section)
Provide DOI OR other citation details if **external detailed step-by-step protocols** are available.	Yes	Materials and Methods

Experimental study design and statistics	Information included in the manuscript?	In which section is the information available? (Reagents and Tools Table, Materials and Methods, Figures, Data Availability Section)
Include a statement about **sample size** estimate even if no statistical methods were used.	Yes	Figures and associated legends, Materials and Methods
Were any steps taken to minimize the effects of subjective bias when allocating animals/samples to treatment (e.g. **randomization procedure**)? If yes, have they been described?	Not Applicable	
Include a statement about **blinding** even if no blinding was done.	Not Applicable	
Describe **inclusion/exclusion criteria** if samples or animals were excluded from the analysis. Were the criteria pre-established?<br><br>If sample or data points were omitted from analysis, report if this was due to attrition or intentional exclusion and provide justification.	Not Applicable	
For every figure, are **statistical tests** justified as appropriate? Do the data meet the assumptions of the tests (e.g., normal distribution)? Describe any methods used to assess it. Is there an estimate of variation within each group of data? Is the variance similar between the groups that are being statistically compared?	Not Applicable	

Sample definition and in-laboratory replication	Information included in the manuscript?	In which section is the information available? (Reagents and Tools Table, Materials and Methods, Figures, Data Availability Section)
In the figure legends: state number of times the experiment was **replicated** in laboratory.	Yes	Figures legends
In the figure legends: define whether data describe **technical or biological replicates**.	Yes	Figures legends

Ethics

Ethics	Information included in the manuscript?	In which section is the information available? (Reagents and Tools Table, Materials and Methods, Figures, Data Availability Section)
Studies involving **human participants**: State details of **authority granting ethics approval** (IRB or equivalent committee(s), provide reference number for approval.	Not Applicable	
Studies involving **human participants**: Include a statement confirming that **informed consent** was obtained from all subjects and that the experiments conformed to the principles set out in the WMA Declaration of Helsinki and the Department of Health and Human Services Belmont Report.	Not Applicable	
Studies involving **human participants:** For publication of **patient photos**, include a statement confirming that consent to publish was obtained.	Not Applicable	
Studies involving experimental **animals**: State details of **authority granting ethics approval** (IRB or equivalent committee(s), provide reference number for approval. Include a statement of compliance with ethical regulations.	Not Applicable	
Studies involving **specimen and field samples:** State if relevant **permits** obtained, provide details of authority approving study; if none were required, explain why.	Not Applicable	

Dual Use Research of Concern (DURC)	Information included in the manuscript?	In which section is the information available? (Reagents and Tools Table, Materials and Methods, Figures, Data Availability Section)
Could your study fall under dual use research restrictions? Please check biosecurity documents and list of **select agents and toxins** (CDC): https://www.selectagents.gov/sat/list.htm	Not Applicable	
If you used a select agent, is the security level of the lab appropriate and reported in the manuscript?	Not Applicable	
If a study is subject to dual use research of concern regulations, is the name of the **authority granting approval and reference number** for the regulatory approval provided in the manuscript?	Not Applicable	

Reporting

The MDAR framework recommends adoption of discipline-specific guidelines, established and endorsed through community initiatives. Journals have their own policy about requiring specific guidelines and recommendations to complement MDAR.

Adherence to community standards	Information included in the manuscript?	In which section is the information available? (Reagents and Tools Table, Materials and Methods, Figures, Data Availability Section)
State if relevant guidelines or checklists (e.g., **ICMJE, MIBBI, ARRIVE, PRISMA**) have been followed or provided.	Not Applicable	
For **tumor marker prognostic studies,** we recommend that you follow the **REMARK** reporting guidelines (see link list at top right). See author guidelines, under 'Reporting Guidelines'. Please confirm you have followed these guidelines.	Not Applicable	
For **phase II and III randomized controlled trials**, please refer to the **CONSORT** flow diagram (see link list at top right) and submit the CONSORT checklist (see link list at top right) with your submission. See author guidelines, under 'Reporting Guidelines'. Please confirm you have submitted this list.	Not Applicable	

Data Availability

Data availability	Information included in the manuscript?	In which section is the information available? (Reagents and Tools Table, Materials and Methods, Figures, Data Availability Section)
Have **primary datasets** been deposited according to the journal's guidelines (see 'Data Deposition' section) and the respective accession numbers provided in the Data Availability Section?	Yes	Materials and Methods, Data Availability Section
Were **human clinical and genomic datasets** deposited in a public access-controlled repository in accordance to ethical obligations to the patients and to the applicable consent agreement?	Not Applicable	
Are **computational models** that are central and integral to a study available without restrictions in a machine-readable form? Were the relevant accession numbers or links provided?	Yes	Materials and Methods, Data Availability Section
If publicly available data were reused, provide the respective **data citations in the reference list**.	Yes	Figures legends, Materials and Methods, Reference list
